# Expanding the Genetic and Clinical Spectrum of *SCN1A*-Related Hemiplegic Migraine: Analysis of Mutations in Japanese

**DOI:** 10.3390/ijms26041426

**Published:** 2025-02-08

**Authors:** Daisuke Danno, Haruka Tada, Itsuki Oda, Norihito Kawashita, Makito Hirano, Shigekazu Kitamura, Shoji Kikui, Makoto Samukawa, Keisuke Yoshikawa, Yoshiyuki Mitsui, Yoshitaka Nagai, Takao Takeshima, Kazumasa Saigoh

**Affiliations:** 1 Headache Center, Department of Neurology, Tominaga Hospital Japan, Osaka 556-0017, Japan; daisukedanno@yahoo.co.jp (D.D.); kikui@tominaga.or.jp (S.K.); ttakeshi@tominaga.or.jp (T.T.); 2 Department of Life Science, Faculty of Science and Engineering, Kindai University, Osaka 577-8502, Japan; t.haruka1242@gmail.com (H.T.); 105w.lvnnjowh@gmail.com (I.O.); nkawashita@emat.kindai.ac.jp (N.K.); 3 Department of Clinical Genetics, Kindai University Hospital Japan, Osaka 589-8511, Japan; 4 Department of Neurology, Faculty of Medicine, Kindai University, Osaka 589-8511, Japan; mahirano-neuro@umin.ac.jp (M.H.); daruma@med.kindai.ac.jp (M.S.); garvsx9s@med.kindai.ac.jp (K.Y.); mitsui@med.kindai.ac.jp (Y.M.); yoshi.nagai@med.kindai.ac.jp (Y.N.); 5 Department of Neurology, Konan Medical Center, Kobe 658-0064, Japan; kit_kakkun@yahoo.co.jp

**Keywords:** sporadic hemiplegic migraine (SHM), familial hemiplegic migraine (FHM), *CACNA1A*, *ATP1A2*, *SCN1A*, Dravet syndrome

## Abstract

Familial hemiplegic migraine (FHM) is characterized by repeated episodes of reversible localized neurological deficits, in addition to headache. The aura of HM includes visual, sensory, motor, and verbal symptoms. Hemiplegic migraine (HM) is classified into non-familial sporadic HM (SHM) and familial HM (FHM). Here, we analyzed the clinical symptoms and their relevance in four Japanese patients considered to have *SCN1A* mutations as a cause. Sequencing of *SCN1A* was performed using a whole exome sequence method in 48 blood samples from clinically suspected patients with FHM. Subsequently, algorithm analysis, allele frequency determination, and three-dimensional structure analysis of the recognized variants were performed, and the recognized variants were evaluated. We found five heterozygous missense mutations (p.A23E, p.V250L, p.T398M, p.R1575C, p.L1660I) in *SCN1A*, three of which had not been reported. These five mutations may also affect the structure of the protein products, as assessed using a three-dimensional structural analysis. In all cases, the clinical symptoms included visual, sensory, motor, and verbal symptoms, which are forms of aura. Similarities were detected, such as the appearance of symptoms at a young age and other symptoms, such as hemiplegia after a headache attack. We report five missense mutations in *SCN1A* of Japanese cases.

## 1. Introduction

Migraine has a high prevalence rate of 8.4% in Japan, and the high proportion of patients in the working-age group causes significant socioeconomic losses [1]. Despite this, the underlying causes and pathogenesis of migraine remain largely unknown. One clue to elucidating the pathomechanism of migraine is to study the genes that cause migraine. Migraine is characterized by two main types: migraine without aura (MO) and migraine with aura (MA) [2]. Among migraine with aura, familial hemiplegic migraine, in particular, is a subtype of migraine that presents with headache after an aura of hemiplegia [3].

Currently, the most well-known migraine genes are FHM1, FHM2, and FHM3, which are known as familial hemiplegic migraine (FHM). Hemiplegic migraine (HM) is classified as one of the migraine headaches with aura [3]. Those with a family history of migraine, in particular, are referred to as familial hemiplegic migraine. Currently, there are three major types of the gene, *CACNA1A*, *ATP1A2*, and *SCN 1A* [4,5].

The *CACNA1A* is known not only as the causative gene for FHM type 1, but also for episodic ataxia type 2 (EA type 2) [6,7]. Hemiplegic migraine type 1 is characterized by headaches complicated by cerebellar symptoms such as “lightheadedness” and “dizziness”, sometimes accompanied by brainstem symptoms. On the other hand, in EA type 2, its clinical manifestation is usually a rare disease with paroxysmal ataxia, usually not accompanied by headache [6].

FHM2 (*ATP1A 2*) is the most frequent of the FHMs. Its analysis is also in progress: in FHM2, there are four sodium pump α subunit genes present during its attacks, three of which (α1, α2, and α3) are expressed in the central nervous system, and α2 is α2 is specific to glial cells. Based on cerebral blood flow scintigraphy in Japanese, it is thought to be able to reproduce CSD, and some of its mutations showed a preventive effect on lomerizine [8,9].

FHM3 caused by *SCN1A* is considered epidemiologically less frequent than other FHMs. *SCN1A* encodes the α1 subunit of the neuronal voltage-gated sodium (Nav1.1), which mediates the voltage-dependent sodium ion permeability of excitable membranes of the CNS. Voltage-gated sodium channels play a major role in neuronal excitation. Sodium channels are composed of an α-subunit, which is the main subunit that forms pores (holes through which ions pass), and a β-subunit that regulates the opening and closing of the pore. The α-subunit is expressed in different tissues and organs [10,11].

Other hand, Dravet syndrome of severe epilepsies is almost caused by *SCN1A* mutation [12,13]. These *SCN1A* mutations are caused by epileptic disorders that are more major than FHM3. Generalized epilepsy with febrile seizures plus 2 (GEFS+2) is a rare autosomal dominant, familial condition with incomplete penetrance and large intrafamilial variability. Patients display febrile seizures persisting sometimes beyond the age of 6 years and/or a variety of afebrile seizure types [13]. This disease combines febrile seizures, generalized seizures often precipitated by fever at age 6 years or more, and partial seizures, with a variable degree of severity [14,15,16].

In this study, we aimed to report our findings on *SCN1A* mutations causing FHM2 type, its phenotype, and treatment in Japanese patients.

## 2. Results

### 2.1. The Whole Exome Sequence Analysis of SCN1A

**Figure 1:**
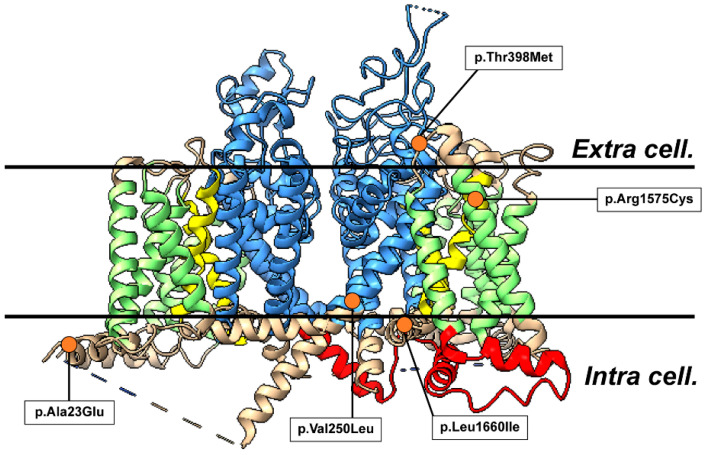
**Structural model of the *SCN1A* protein and point mutation positions**. The three-dimensional structure of *SCN1A* is transmembrane-domains protein in the MOE program. The protein consists of four homologous domains (D1–4) each formed of six transmembrane segments (S1–S6). Segment 4 represents the voltage sensor and segments 5–6 the pore region. The transmembrane domains are indicated in green, the actuator domain is indicated in blue, the phosphorylation-binding domain is indicated in yellow, and the nucleotide-binding domain is indicated in red.. The mutation positions in our five cases is shown by the orange dots.

Sequencing of *SCN1A* was performed using the exome sequencing method in 48 blood samples from clinically suspected patients with familial HM. We detected five heterozygous missense mutations c.68C>A (p.Ala23Glu) of No. 1 patient, c.748G>C (p.Val250Leu) of No. 2, c.1193C>T (p.Thr398Met) of No. 3, c.4723C>T (p.Arg1575Cys) of No. 4, and c.4979C>A (p.Leu1660Ile) of No. 5 in *SCN1A*. Three variants(p.Ala23Glu, p.Val250Leu, and p.Leu1660Ile) had not been reported in the ClinVar database (Figure 1).

**Figure 2:**
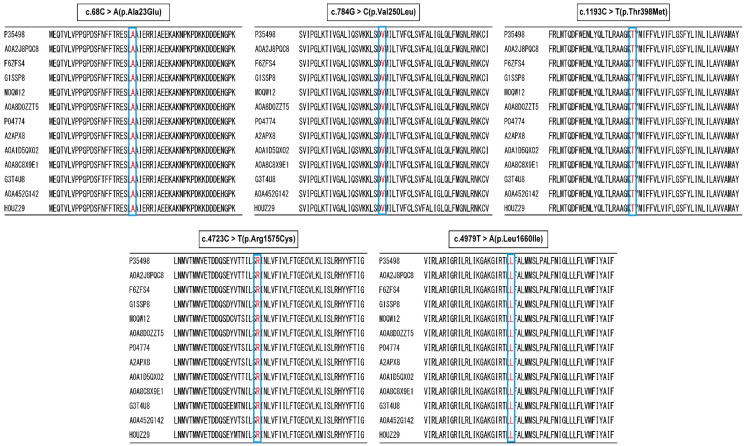
**Amino acid sequence alignment of the five mutations.** The positions of the five mutations in *Homo sapiens* are highly conserved across species, amino acid in blue box, including humans (*Homo sapiens*; NP_001159435.1; P35498), Chimpanzee (*Pan troglodytes*; XP_009441904.1; A0A2J8PQC8), Horse (*Equus caballus*: ENSECAT00000019779.3; F6ZFS4), Rabbit(*Oryctolagus cuniculus*; XP_002712295.1; G1SSP8), Bovine (*Bos taurus*; XP_015330784.1; M0QW12), Pig (*Sus scrofa*; ENSSSCT00035061042.1; A0A8D0ZZT5), Rat (*Rattus norvegicus*; NP_110502.1; P04774), Mouse (*Mus musculus*; NP_001300926.1; A2APX8), Rhesus macaque (*Macaca mulatta*; XP_001101023.1; A0A1D5QX02), Lion (*Panthera leo*; ENSPLOT00000015459.1; A0A8C8X9E1), African elephant (*Loxodonta Africana*; XP_003405863.1; G3T4U8), Goat (*Capra hircus*; ENSCHIT00000038215.1; A0A452G142), and Guinea pig (*Cavia porcellus*; ENSCPOT00000002700.3; H0UZ29). The sequences were aligned using the NCBI homologene website (http://www.ncbi.nlm.nih.gov/homologene (accessed on 6 May 2024)).

Moreover, all mutation sites were highly conserved across species in many mammals of Amino acid sequence alignment (Figure 2). The mutations were predicted to lead to the formation of a new salt bridge and of a protein turn around the positive-charged residue. Moreover, these two mutations were not represented in the 1000 Genomes database of the Japanese (https://ijgvd.megabank.tohoku.ac.jp (accessed on 6 May 2024)) or the NCBI ClinVer site (https://www.ncbi.nlm.nih.gov/clinvar/ (accessed on 6 May 2024)).

### 2.2. Allele Frequency and In Silico Analysis

The allele frequencies for East Asians were confirmed by NCBI: No. 1 was 0.0000, No. 2 and No. 5 were not reported, No. 3 was 0.00, and No. 4 was 0.0022. The allele frequencies for Japanese were confirmed by jMorp: No. 1, 2, and 5 were not reported, No. 3 was 0.000646, and No. 4 was 0.0022. The allele frequencies of No. 1, 2, and 5 were not reported from jMorp, No. 3 was 0.000646, and No. 4 was 0.010001. Variant prediction analysis of the seven algorithms yielded the results shown in Table 1. From the abnormalities, the five variants found in this study were judged to be mutations in *SCN1A*.

We analyzed the pathogenic mutation prediction in five software programs (SIFT version 4.0.3, PolyPhen2 version 2.2.3, MutationTaster2 version 2021, PROVEAN version 1.1.3, PANTHER 19.0, CADD version 1.7, and M-CAP version 1.4.0). Red typed recognised as pathogenic.

### 2.3. Three-Dimensional Structural Analysis

**Figure 3:**
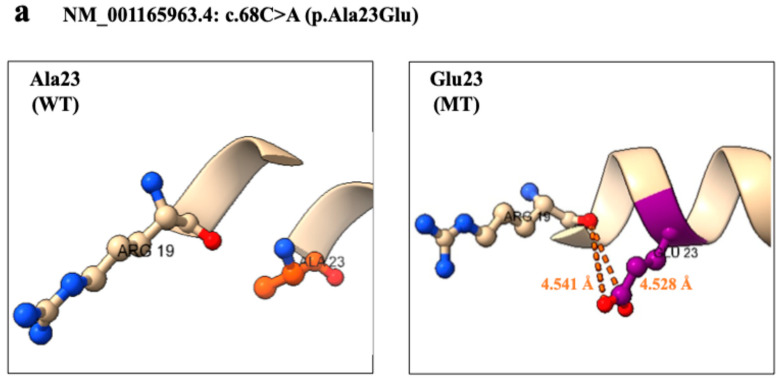

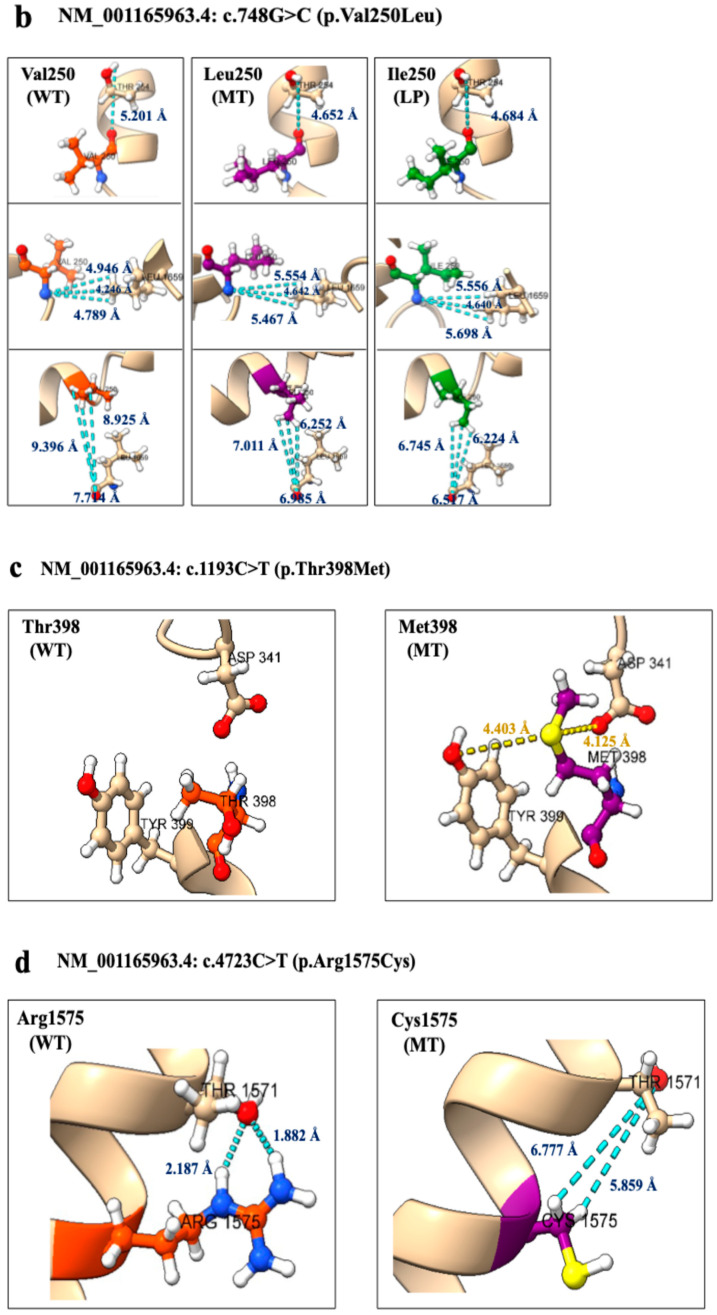

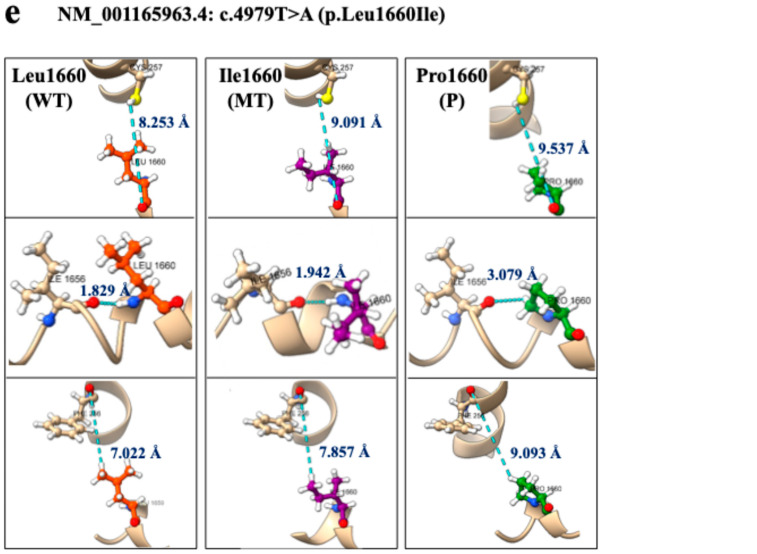
**Three-Dimensional Structural Analysis of five mutation.** (**a**) **NM_001165963.4: c.68C>A (p.Ala23Glu) (rs13939722).** No. 1 mutation position was present at the site forming an α-helix in the intracellular loop structure of *SCN1A* domain I. Comparing the wild type (WT) and the mutant type (MT), the number of peripheral residues within 5 Å of the mutant increased by one residue. In addition, the No. 1 variant was a change from a hydrophobic to a hydrophilic residue, confirming a change in the hydrophobic environment and a change in the volume of the side chain. (**b**) **NM_001165963.4: c.748G>C (p.Val250Leu).** No. 2 was located in S5 of the pore-forming region of domain I. Comparing the wild-type and mutant forms of No. 2, the number of peripheral residues within 5 Å increased by 2. Hydrogen bonds were observed between Val250 and Thr254 and between Leu250 and Thr254, but at equivalent distances of 1.796 Å and 1.836 Å, respectively. Structural changes were observed in the mutant compared to the wild type, such as the α-helix changing to a loop structure and the loop structure changing to an α-helix. Furthermore, Ile250, with the same site substitution, has been reported as likely to be pathogenic (LP); comparison of the modeling structure of Leu250 and Ile250 revealed changes in peripheral structure at 9 residues similar to those of Ile250. (**c**) **NM_001165963.4: c.1193C>T (p.Thr398Met) (rs774937055).** No. 3 was located in the extracellular membrane pore of domain I. Comparison of the wild type and the mutant showed that the number of peripheral residues within 5 Å of the mutant was increased by 2 residues. Confirming the interaction between the variant site and the surrounding residues, hydrogen bonds were observed between Thr398 and Asp341, and Met398 and Asp341, but at comparable distances of 1.788 Å and 1.794 Å, respectively. New S-O interactions were observed at Ser339, Asp341, and Tyr399, with distances of 7.041 Å, 4.125 Å, and 4.403 Å, respectively. Compared to the wild type, the mutant Ser339 was pulled toward the intracellular side, and a large conformational change was observed. (**d**) **NM_001165963.4: c.4723C>T (p.Arg1575Cys) (rs121918807).** In WT, hydrogen bonds with Thr1571 were observed in two locations. On the other hand, **in MT, the distance of the hydrogen bond was elongated, and the effect of the hydrogen bond was weakly modified.** (**e**) NM_001165963.4: c.4979T>A (p.Leu1660Ile). Comparing WT and MT, the orientation of the side chain of Cys257 was changed. In addition, MT showed a change in the distance of interaction and peripheral structure similar to that of Pro1660. The Pro1660 substitution at the same site was reported to be pathogenic (P) by ClinVar, and the present mutation was also judged to be pathognomonic mutation.

No. 1 was present at the site forming an α-helix in the intracellular loop structure of SCN1A domain I. Comparing the wild type (WT) and the mutant type (MT), the number of peripheral residues within 5 Å of the mutant increased by one residue. In addition, the No. 1 variant was a change from a hydrophobic to a hydrophilic residue, confirming a change in the hydrophobic environment and a change in the volume of the side chain (Figure 3a).

No. 2 was located in S5 of the pore-forming region of domain I. Comparing the wild-type and mutant forms of No. 2, the number of peripheral residues within 5 Å increased by 2. Hydrogen bonds were observed between Val250 and Thr254 and between Leu250 and Thr254, but at equivalent distances of 1.796 Å and 1.836 Å, respectively (Figure 3b). Structural changes were observed in the mutant compared to the wild type, such as the α-helix changing to a loop structure and the loop structure changing to an α-helix. Furthermore, Ile250, with the same site substitution, has been reported as likely to be pathogenic; the comparison of the modeling structure of Leu250 and Ile250 revealed changes in peripheral structure at 9 residues similar to those of Ile250.

No. 3 was located in the extracellular membrane pore of domain I. Comparison of the wild type and the mutant showed that the number of peripheral residues within 5 Å of the mutant increased by 2 residues. Confirming the interaction between the variant site and the surrounding residues, hydrogen bonds were observed between Thr398 and Asp341, and Met398 and Asp341, but at comparable distances of 1.788 Å and 1.794 Å, respectively (Figure 3c). New S-O interactions were observed at Ser339, Asp341, and Tyr399, with distances of 7.041 Å, 4.125 Å, and 4.403 Å, respectively. Compared to the wild type, the mutant Ser339 was pulled toward the intracellular side, and a large conformational change was observed. Furthermore, the No. 3 variant was a change from hydrophilic to hydrophobic residues, confirming a change in the hydrophobic environment and a change in the volume of the side chain.

No. 4 was present in S2 of domain IV. Comparison of the wild type and the mutant showed that the number of peripheral residues within 5 Å was reduced by one residue, and the interaction between the variant site and the peripheral residues was confirmed by ChimeraX and MOE, showing that hydrogen bonds with the side chain of Thr1571 were observed at distances of 1.882 Å and 2.187 Å in the wild type, but these hydrogen bonds disappeared in the mutant. Hydrogen bonds were lost in the mutant. Other hydrogen bonds were also observed in the main chains of Arg1575 and Thr1571 and Cys1575 and Thr1571, but at comparable distances of 1.865 Å and 1.847 Å, respectively. Hydrogen bonds were also observed in Arg1575 and Val1579, and Cys1575 and Val1579, with comparable distances of 2.049 Å and 1.979 Å, respectively (Figure 3d). Furthermore, structural changes were observed, such as changes in the volume of the side chains and the orientation of the side chains around the variant site.

No. 5 was present in the cell between S4 and S5 of domain IV. Comparing the wild type and the variant, the number of peripheral residues within 5 Å increased by one residue and decreased by one residue. Confirming the interaction between the variant site and the surrounding residues, no new interactions were formed or lost; hydrogen bonds were observed between Leu1660 and Arg1657, and Ile1660 and Arg1657, but at comparable distances of 2.266 Å and 2.299 Å, respectively. Hydrogen bonds were also observed between Leu1660 and Ile1656 and Ile1660 and Ile1656, but at comparable distances of 1.829 Å and 1.942 Å, respectively (Figure 3e). Structural changes were observed in the mutant compared to the wild type, such as the α-helix changing to a loop structure and the loop structure changing to an α-helix. In addition, the same site substitution, Pro1660, has been reported to be pathogenic; comparison of the modeling structures of Ile1660 and Pro1660 revealed changes in the peripheral structure at 10 residues similar to those of Pro1660.

### 2.4. Five Variants Classification

The No. 1 variant was reported as a variant of uncertain significance (VUS) in the Clinver database. But in this variant is an extremely low allele frequency in East Asians. In silico analysis predicted pathogenicity for 6/7 algorithms. Conservation of the amino acid sequence was confirmed across four mammalian species identical to humans. Protein modeling localized the No. 1 variant to an α-helical domain I intracellular loop, potentially disrupting the structure. As the No. 1 patient experienced childhood seizures, a causal role cannot be excluded. Integrating these data as per ACMG guidelines, the No. 1 variant met the criteria for being likely pathogenic. Similarly, when analyzed, No. 2 satisfied the likely pathogenic criteria, and No. 3 matched the likely pathogenic criteria. No. 4 was reported as a variant of VUS. The structure of the variant was influenced by changes in the volume of the side chains and the orientation of the side chains around the variant site, and it was finally judged to be a pathological mutation. No. 5 satisfied the likely pathogenic criteria, congruent with its likely detrimental effects. In summary, applying stringent variant curation protocols informed causal inferences for these *SCN1A* variants identified in our epileptic cohort. Further functional validations are still warranted.

### 2.5. Clinical Features of Patients

In a clinical and genetic mutation association study, the five patients in whom missense mutations were detected shared common migraine symptoms with typical aura, including visual, sensory, motor, and speech symptoms, as well as dizzy attacks, in addition to headache and paralysis. Among them, visual symptoms, such as visual field constriction and light perception, were most common. First attacks were symptomatic from less than 20 years of age (Table 2). On the other hand, the frequency of headache attacks and the duration and frequency of paralysis differed from case to case. However, no differences in clinical symptoms due to genetic mutations were observed (Supplementary Data). In addition, each case had undergone the brain MRI scan, which showed no abnormal findings. There were also no neurological findings at any time other than during the headache attacks.

## 3. Discussion

We performed whole exome sequencing of *SCN1A* in a proband presenting with headache. Five missense variants were detected within *SCN1A* and subjected to further investigation. Standardized pathogenicity assessment involved searching public allele frequency databases and applying in silico predictive algorithms to estimate deleteriousness. Sequence conservation was determined across evolutionarily divergent species to infer evolutionary constraints. Protein structural modeling provided insight into potential functional consequences at the tertiary structure level.

Two variants (p. Ala23Glu of No. 1 patient and p.Val250Leu of No. 2) were classified as likely pathogenic and three variants as a VUS (p.Thr398Met of No. 3, p.Arg1575Cys of No. 4, and p.Leu1660Ile of No. 5) according to the previous Clinver database. Three variants (No. 3, No. 4, and No. 5) were predicted to be pathogenic based on structural modeling (Table 1 and Figure 3). As our data, to summarize the present study, five pathological mutations were observed. While public database searches and structural analyses provide useful insights, comprehensive variant curation requires the integration of family history data and reported cases. Further evaluations including co-segregation analysis in biologically related family members as well as functional assays at the cellular level, such as measurements of ion channel currents, will strengthen causal inferences for identified variants. Applying multi-tiered variant assessment strategies offers the best approach for elucidating genotype–phenotype relationships.

The results of the clinical investigation showed that most of the patients in whom the missense variant in *SCN1A* was detected had migraine symptoms with typical aura, such as visual, sensory, motor, and speech symptoms, and dizzy attacks, in addition to headache and paralysis. Among them, visual symptoms such as visual field constriction and light perception were found to be common in the present patients. In addition, clinical symptoms commonly appeared in adolescents under 20 years of age. On the other hand, the frequency of headache attacks and the duration and frequency of paralysis experienced differed from case to case. We attempted to clarify the differences in clinical symptoms among the variants, but no clear relationship between the five missense variants and clinical symptoms could be confirmed. In the present genetic analysis, we found that some cases with sporadic genetic mutations were found even in the three cases with no family history of the disease. This result suggests that there may be an unexpectedly large number of cases showing de novo mutations in SCA1A, even in cases with no family history. Therefore, cases with hemiplegia or unilateral sensory impairment should be aggressively tested.

Consider the relationship between SHM/FHM and clinical symptoms. The study of severe forms of epilepsy and variants in Dravet syndrome in genetic studies is instructive. The gene is also known as the gene for Dravet syndrome, a severe form of childhood epilepsy in which the primary seizure is usually a unilateral or generalized clonic or tonic–clonic seizure with or without fever within the first year of life [17]. Subsequently, genetic analysis of patients with Dravet syndrome at multiple centers revealed that approximately 80% of the patients had *SCN1 A* gene mutations [18,19]. Dravet syndromes with *SCN1 A* mutations show a wide spectrum of phenotypes ranging from benign with spontaneous remission to refractory with fatal consequences.

Furthermore, patch–clamp analysis of the electrophysiological properties of mutant channel proteins in Dravet syndromes associated with *SCN1 A* mutations reveals both gain- and loss-of-function phenotypes [20], and the correlation with clinical symptoms is unclear. However, in more severe phenotypes, including severe myoclonic epilepsy of infancy (SMEI) and SMEI-borderline (SMEB), the rate of missense mutations is decreasing. Missense mutations identified in severe phenotypes occurred more frequently in the pore region of Nav1.1 than those underlying milder phenotypes [21,22]. Indeed, in our SHM/FHM studies, few of the proteins in the transmembrane region of the channel, even when present in the transmembrane region, resulted in major protein mutations based on their 3D structure [23,24].

Thirdly, it has been shown that the severity of orchitis in *Scn1a* knockout mice is greatly influenced by the genetic background of the strain [25]. Nav1.1 is predominantly expressed in the inhibitory GABAergic nervous system compared to the excitatory glutamatergic nervous system.

Therefore, patients with *SCN1A* gene mutations are thought to have impaired Nav1.1 function (impaired Na+ passage), resulting in reduced function of inhibitory signaling and epileptic seizures [26,27]. If an alteration in *SCN1A* is identified in a patient that results in a more severe alteration of the *SCN1A* protein (e.g., a frameshift or nonsense mutation that results in a shortening of the protein), the patient is more likely to have a more severe symptomatic SMEI or SMEI-related syndrome rather than SHM or FS symptoms [23,24,25]. These *SCN1A*-related Dravet syndrome disorders lie at different ends of the *SCN1A*–disease spectrum, reflecting the degree of a loss of function in inhibitory interneurons. Given the underlying loss of function disease mechanism, individuals typically experience seizure exacerbation following sodium channel blocker (SCB) use [23]. By contrast, gain-of-function *SCN1A* variants are associated with FHM3. Individuals present with a severe subtype of migraine with aura characterized by hemiparesis during the attacks.

Administration of anti-seizure medication that strongly inhibit Na^+^ channels in patients with Dravet syndrome leads to exacerbation of seizures [28,29]. The reason for this is that *SCN1A* seizure disorder is inherited in an autosomal dominant manner, so probands with *SCN1A* seizure disorder may have hereditary or de novo pathogenic variants. The proportion of cases with de novo pathogenic variants depends on the phenotype: parents affected with *SCN1A* seizure disorder, and the proportion of probands with the proband phenotype decreases with increasing severity of the proband phenotype. Thus, most cases of *SCN1A*-associated pediatric severe myoclonic epilepsy (*SCN1A*-SMEI) and ICE-GTC are the result of de novo pathogenic variants [30].

On the other hand, patients with SHM/FHM have milder clinical symptoms than those with DS and are therefore able to carry offspring. Thus, mutations in *SCN1A* are often passed on to offspring.

This study investigated *SCN1A* gene mutations and clinical symptoms in Japanese migraine patients with FHM type 3. At the same time, we examined the form of *SCN1A* mutations based on previous studies in Dravet syndrome and found that they were in the form of missense mutations, with no major genetic mutations such as truncation mutations or deletion mutations. Now, it is clearer that FHM type 3 and Dravet syndrome are genetically close disease concepts. In the future, we hope to develop new treatments based on the genetic homology between FHM type 3 and Dravet syndrome.

## 4. Methods

### 4.1. Genome DNA Extraction from Peripheral Blood

Blood samples of 48 patients were collected from adults who understood the nature of the study and provided a consent agreement. Peripheral blood was anticoagulated with sodium heparin and extracted using a QIAGEN DNA Blood Mini Kit (QIAGEN, Hilden, Germany). First, 20 µL of Protease K and lysis buffer (Buffer AL 200 µL) were added to 200 µL of whole blood and incubated at 56 °C for 15 min. Then, 200 µL of 100% ethanol was added, transferred to a spin column, and centrifuged to adsorb the DNA onto the column. The column was washed with two different wash buffers (Buffer AW1 500 µL, Buffer AW2 500 µL), and DNA was extracted with an elution buffer (Buffer AE 100 µL).

### 4.2. The Whole Exome Sequence of SCN1A

Screening of the *SCN1A* genes was performed using a sequencing analysis. Sequencing reagents were a NovaSeq6000 S4 Reagent Kit (Illumina, Inc., San Diego, CA, USA) and NovaSeq Xp 4-Lane Kit, and instruments were NovaSeq6000 (Illumina, Inc.) for detection. The software NovaSeq Control Software vl.7.0 and Real-Time Analysis v3.4.4 were used. Read sequences obtained via the sequencing analysis were mapped to the genome sequence. Duplicate reads were marked from the mapping results, and the 500 bp before and after the SureSelect target region was targeted to detect candidate variant nucleotides that differ from the reference sequence. The detected variant bases were then filtered, and the following annotation information was added using SnpEff and Vcftools. Mutations found via exome analysis were confirmed using direct sequencing.

### 4.3. Direct Sequencing of SCN1A

Mutations found via whole exome analysis were confirmed using direct sequencing. PCR products and primers were mixed and sequenced. In total, 1.0 μL of a BigDye terminator ver. 3.1 Cycle Sequence Mix and 1.5 μL of a 5× Sequencing Buffer were added to each tube to make 10 μL. A total of 10 s at 96 °C and 5 s at 50 °C for 30Cycle and 60 °C for 4 min Cycle sequencing was performed. Ten microliters of the reacted sample was mixed with 125 mM EDTA (pH 8.0) and 100% ethanol, left at room temperature in the dark for 15 min, and centrifuged (room temperature, 8600 rpm, 5 min). The supernatant was removed by flushing, and 100 μL of 70% ethanol was added and centrifuged (room temperature, 8600 rpm, 5 min). The supernatant was removed by flushing. A total of 10 μL of HiDi was added and left in the dark for 20 min to dissolve (96 °C, 3 min for reaction, and analyzed using ABI PRISM 3130XL or ABI PRISM 3500XL of Thermo Fisher Scientific Inc., Waltham, MA, USA).

### 4.4. Allele Frequency and Variant Prediction Analysis (In Silico Analysis)

A SIFT (Sorting Intolerant From Tolerant) SNP function prediction algorithm analysis first converts the variant list into a VCF format that can be used by SIFT. Coding SNVs fail to save the file, update the file for SNPs, and perform the analysis. For PolyPhen2, the protein registration number was entered in the protein or SNP identifier for PolyPhen2. If the SNP registration number was available, the number was entered in the protein or SNP identifier. For substitution, the amino acid substitution location (codon position) was entered, and the amino acid before amino acid substitution was selected in the upper row, and the amino acid after amino acid substitution was selected in the lower row for analysis [7,8,9]. The mutation was entered in the alteration field. PROVEAN was used to enter the gene name and protein reference sequence first. For PANTHER, mutant sequences were entered, DI List was selected, microarray IDs were converted to Ensemble IDs, and Homo sapiens was selected and analyzed. CADD (Combined Annotation Dependent Depletion) results are presented as score only, but reports suggest pathogenicity if above 20.0 [31,32]. M-CAP (The Mendelian Clinically Applicable Pathogenicity) score is a pathogenicity likelihood score that aims to misclassify no more than 5% of pathogenic variants while aggressively reducing the list of variants of uncertain significance [33].

### 4.5. Three-Dimensional Structural Analysis

The crystal structure of the human voltage-gated Na+ channel (7DTD) was obtained from the Protein Data Bank (PDB), and the template structure was created using the Molecular Operating Environment (MOE). The three-dimensional structure of the variant was modeled based on the template structure. For modeling of variant sites for which three-dimensional structures had not been established, LocalColabFold was used. Changes in the surrounding structures and interactions between the wild-type and mutant-modeling structures were analyzed using ChimeraX. For some variants, the structures of the variants detected in this study were compared to those of variants with the same site substitution for which virulence evaluations have already been reported and analyzed.

### 4.6. Variant Classification of ACMG Guidelines

It is necessary to evaluate whether variants detected in genetic analysis actually cause disease. The American College of Medical Genetics (ACMG), Association of Molecular Pathology (AMP), and College of American (CAP) jointly published guidelines [34]. The ACMG guidelines are characterized by evaluating all variants according to multiple criteria for pathogenicity and then synthesizing each evaluation to determine the final pathogenicity.

## Figures and Tables

**Table 1:** Variant prediction analysis (in silico analysis).

	c.68T>A (p.Ala23Glu)	c.748G>C (p.Val250Leu)	c.1193C>T (p.Thr398Met)	c.4723C>T (p.Arg1575Cys)	c.4979T>A (p.Leu1660Ile)
**Mutation Taster**	**Disease causing** **(107)**	**Disease causing** **(32)**	**Disease causing** **(81)**	Polymorphism −180	**Disease causing** **(99)**
**PolyPhen-2**	**Possibly damaging** **−0.744**	**Possibly damaging** **−0.903**	**Possibly damaging** **−0.989**	**Possibly damaging** **−1**	**Possibly damaging** **−1**
**PROVEAN**	**Deleterious** **(−0.760)**	**Deleterious** **−2.931**	**Deleterious** **(−4.215)**	Neutral (−1.973)	Neutral (−1.742)
**SIFT**	Predict tolerated −0.28	**Predict not tolerated** **−0.01**	Predict tolerated −0.34	Predict tolerated −0.18	**Predict not tolerated** **−0.01**
**PANTHER**	**Probably damaging** **−0.74**	**Probably damaging** **−0.85**	**Probably damaging** **−0.74**	**Probably damaging** **−0.5**	**Probably damaging** **−0.85**
**CADD**	**24.5**	**24.9**	**24.8**	**24.3**	**28.4**
**M-CAP**	**Possibly pathogenic** **−0.137**	**Possibly pathogenic** **−0.8717**	**Possibly pathogenic** **−0.789**	**Possibly pathogenic** **−0.177**	**Possibly pathogenic** **−0.9671**

**Table 2:** Clinical characteristics of the five cases.

		No1	No2	No3	No4	No5
Bacic characteristics	Mutation	p.A23E	p.V250L	p.T398M	p.R1575C	p.L1660I
	Age (y)/Sex	29 man	60 woman	23 woman	24 women	54 women
	HM onset (y)	4	20	23	24	14
	Past history	convulsion in 5y	motion sickness in childhood	motion sickness in childhood	motion sickness in childhood	-
	Family history	mother	-	father	-	doughter
	Dominant hand	right	right	right	right	N/A
Aura symptoms during hemiplegic attacks	Hemiplegia	+	+	+	+	+
	Hemiplegia Side	birateral	left dominant	birateral	N/A	left dominat
	sensory disturbance	+	+	+	+	+
	visual disturbance	Light like a tunnel	Fortification	Flickering light, blurred	Scintillation	Jagged light
	aphasia	+	+	+	+	+
	Triggering factors for hemiplegic attacks	stress lack of sleep	atmospheric pressure stress	-	stress, fatigure	crowd of human
	Others	vertigo	vertigo	-	-	vertigo
Duration time and Frequency of attacks	Duration time	intermittent and continuous	5–30 min	10~15 min	30 min	20–30 min
	Frequency	intermittent and continuous	2 times > per year	50 times > per year	5 times > per year	-
Headache attack characteristics	NRS score (0–10)	8–10	2–8	4–6	3–5	2–10
	Duration time	intermittent and continuous	30 min >	2 h >	30 min >	2 h >
	headache day/month	30	20	7–15	10–15	2–10
	Temporal relation to hemiplegic attacks	+	+	+	+	+
	Anatomical relation to hemiplegic attacks	-	-	-	-	-
	Nausea/vomiting	+	+	+	+	+
	photophobia	+	+	+	-	+
	phonophobia	+	-	+	-	5
	Other types of attacks	vertigo, fatigue	vertigo, fatigue	vertigo	-	-

HM: hemiplegic migraine, NRS: numeral rating scale, N/A: not available

## Data Availability

The raw data supporting the conclusions of this article will be made available by the authors on request.

## Supplementary Materials

The following supporting information can be downloaded at: https://www.mdpi.com/article/10.3390/ijms26041426/s1.
