# Supplementary material for: Expanding the Genetic and Clinical Spectrum of SCN1A-Related Hemiplegic Migraine: Analysis of Mutations in Japanese
Source: Int J Mol Sci. 2025 Feb 8;26(4):1426. doi: 10.3390/ijms26041426 (PMC11855537; doi:10.3390/ijms26041426)
Supplement: Supplementary file 1 [file ijms-26-01426-s001.zip › ijms-3439008-supplementary.pdf]

Supplements data

No1 patient holding the p.Ala23Glu variant was a 29-year-old male. The No1 has been tentatively diagnosed with HM, migraine without aura (MO), migraine with aura (MA), and chronic migraine (CM) based on her previous symptoms. The age of onset of headache is 4 years old, and the patient's history includes a seizure at age 5. There is no specific family history for No1. Symptoms from childhood included motion sickness. Migraine attacks with paralysis began to occur when he was 23 years old. Symptoms of childhood included motion sickness. Paralysis persisted for approximately 6 years, with more than 500 hateful episodes. The order of location of symptoms during seizures was as follows: eye symptoms, tremor, headache, numbness symptoms, paralysis, speech symptoms, dizziness, and lightheadedness symptoms. In addition, sometimes the symptoms were feverish for several hours. The Verbal symptoms are loss of speech, and lightheadedness symptoms are decreased level of consciousness.

The p.Val250Leu variant of No. 2 is a 60-year-old female. The patient is no family history with migraine. The diagnosis is HM, MO, and MA. Headache onset is at age 20, with headache attacks occurring with menstruation. As a child, she admitted to motion sickness. And the patient also began to have vertigo complications at age 57. The duration of hemiplegic migraine was 5 minutes in short attacks, 15 minutes in normal attacks, and 30 minutes in long attacks. The number of hemiplegic migraine attacks was more than 100 by the 60th year, and the headaches were accompanied by symptoms of disorientation such as lightheadedness and inability to remember words. The order of symptoms during headache attacks was headache, tingling sensation, hemiplegia, and dizziness/staggering. Numbness symptoms were tingling and tingling sensations.

The patient (No. 3), who retained the p.Thr398Met variant, is a 23-year-old female. Based on her symptoms to date, she was tentatively diagnosed with HM, CM, and MA. The age of onset of headache was 23 years, and she had no specific epileptic symptoms in the past. The patient's father has migraine headaches without aura. Since childhood, the patient has had motion sickness and dizziness. The duration of paralysis associated with migraine headaches is 10 minutes in the shortest case and lasts 30 minutes in the longest case. He has experienced hemiplegic migraine more than 50 times. The order in which symptoms appeared during attacks was: 1 flashes of light, 2 head pain, 3 symptoms of paralysis or numbness, 4 symptoms of dizziness and lightheadedness, and 5 verbal symptoms. The language symptoms included uttering different words than intended, recall of words such as names of objects, and aphasia symptoms in which the patient could not speak when trying to say something.

Patient No. 4, who carried the p.Arg1575Cys variant, is a 24-year-old female. Based on her

symptoms to date, she has been tentatively diagnosed with HM, MA, MO, CM, and medication overuse headache (MOH). The age of onset of migraine headache is 23 years old, and there is no specific history or family history of migraine headache. Symptoms of childhood included motion sickness. Hemiplegic migraine begins with flashes of light followed by the appearance of paralysis. The duration of paralysis is short, less than 15 minutes; normal, between 15 and 30 minutes; and long, lasting 30 minutes. To date, HM has been experienced approximately 5 times.

The patient who carried the p.Leu1660Ile variant of No. 5 is a 23-year-old female. The patient have family history with migraine. Now, she has been tentatively diagnosed with FHM. The age of onset of headache is 13~15 years old, and there is no specific medical history. The patient's family history includes a second daughter who has migraine headaches. Symptoms from childhood included motion sickness. The duration of paralysis and the number of times the patient experienced paralysis, as well as the order in which symptoms appeared during attacks, were unknown because the patient did not respond to these questions. They were aware of symptoms of numbness, such as inability to move their arms and legs well and weakness of the face. They also responded that the symptoms of speech included inability to speak well, difficulty in understanding, and loss of articulation.
